# Advances in electrospun nanofibrous membrane sensors for ion detection

**DOI:** 10.1039/d2ra04911b

**Published:** 2022-12-06

**Authors:** Liangqiang Wu, Yan Song, Shuo Xing, Yapeng Li, Hai Xu, Qingbiao Yang, Yaoxian Li

**Affiliations:** College of Chemistry, Jilin University Changchun 130021 P. R China yangqb@jlu.edu.cn haixu@jlu.edu.cn; College of Materials Science and Engineering, Jilin Institute of Chemical Technology Jilin 132022 P. R. China

## Abstract

Harmful metal ions and toxic anions produced in industrial processes cause serious damage to the environment and human health. Chemical sensors are used as an efficient and convenient detection method for harmful ions. Electrospun fiber membranes are widely used in the field of solid-state chemical sensors due to high specific surface area, high porosity, and strong adsorption. This paper reviews the solid-state chemical sensors based on electrospinning technology for the detection of harmful heavy metal ions and toxic anions in water over the past decade. These electrospun fiber sensors have different preparation methods, sensing mechanisms, and sensing properties. The preparation method can be completed by physical doping, chemical modification, copolymerization, surface adsorption and self-assembly combined with electrospinning, and the material can also be combined with organic fluorescent molecules, biological matrix materials and precious metal materials. Sensing performance aspects can also be manifested as changes in color and fluorescence. By comparing the literature, we summarize the advantages and disadvantages of electrospinning technology in the field of ion sensing, and discuss the opportunities and challenges of electrospun fiber sensor research. We hope that this review can provide inspiration for the development of electrospun fiber sensors.

## Abstract 2

Harmful metal ions and toxic anions produced in industrial processes cause serious damage to the environment and human health.

## Graphical abstract

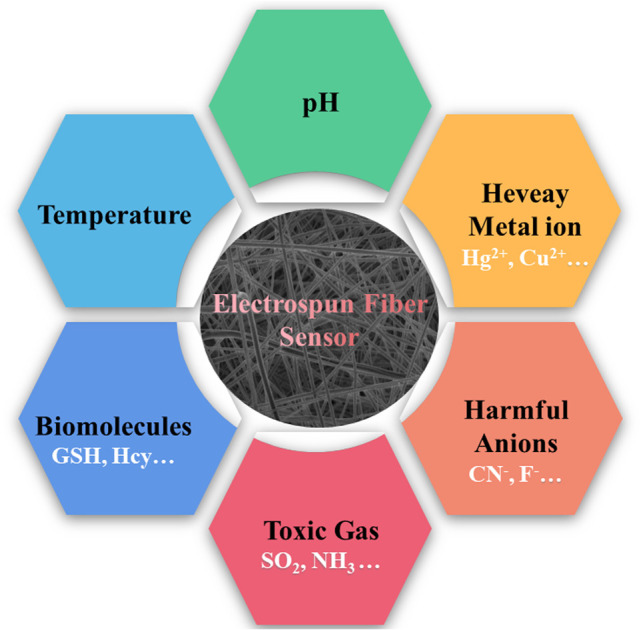

## Introduction

With the development of industry, while bringing progress to human society, it also causes damage to the environment. For example, the harmful metal ions (Cu^2+^, Zn^2+^, Fe^3+^, Al^3+^, Pb^2+^, Hg^2+^) and anions (CN^−^, F^−^, HSO_3_^−^, ClO^−^) in wastewater produced in industrial processes will enter the environment and cause serious health problems to animals and plants in water bodies, including humans.^1–5^

Among them, iron, copper and zinc are essential trace elements for human beings and play vital roles in human physiological activities.^6–9^ However, high levels of them can also affect the normal physiological activities of the body, causing a series of diseases. Excessive intake of Cu^2+^ may cause Alzheimer's disease, familial amyotrophic lateral sclerosis, and Mikan Wilson diseases.^10–12^ Excessive intake of Zn^2+^ can cause poisoning with gastrointestinal symptoms such as vomiting and diarrhea. In addition, it can cause damage to liver, kidney function and immunity.^13–17^ Excessive Fe^3+^ accumulate in body can lead to tissue damage, organ failure and eventually death.^18–20^ Excessive aluminum ions in the body can also lead to physiological dysfunction, which may cause mental retardation, Alzheimer's syndrome, *etc.*^21–23^ At the same time, some harmful heavy metal ions (Pb^2+^, Hg^2+^) in the water body will directly cause poisoning to our body. Ingesting trace amounts of harmful heavy metal ions can wreak havoc on our bodies and cause serious diseases such as cancer, cardiovascular disease, brain damage, failure of kidney and nervous system.^24–29^

CN^−^, F^−^ are widely used in metallurgy and production of daily chemicals. However, cyanide will rapidly combine with iron in cytochrome oxidase, inhibiting the activity of oxidase, resulting in the inability of cells to use oxygen, causing symptoms such as vomiting, convulsions, loss of consciousness, and even death.^30–32^ Fluoride plays an important role in preventing dental caries and treating osteoporosis, but excessive fluoride can lead to dental fluorosis and bone fluorosis.^33–36^ Hypochlorite is widely used as a common disinfectant and bleach. However, as a kind of reactive oxygen species, it has a significant impact on the physiological activities of the human body. Studies have shown that high levels of hypochlorite in the human body will react with proteins, DNA and RNA to cause various diseases. Such as osteoarthritis, cardiovascular disease, atherosclerosis, pneumonia, pulmonary fibrosis, and even cancer.^37–40^ Sulfur dioxide, sulfite and hydrogen sulfite are common food additives that can be effectively preserved and they also have important physiological roles. For example, SO_2_ can act synergistically with NO to relieve vascular smooth muscle and dilate blood vessels. However, high levels of sulfur dioxide and SO_3_^2−^/HSO_3_^−^ are also toxic. Lung cancer, migraine, stroke and other respiratory, nervous system, cardiovascular and cerebrovascular diseases are all associated with high levels of sulfur dioxide and SO_3_^2−^/HSO_3_^−^.^41,42^

These harmful ions pose a threat to human health. Therefore, it is an inevitable trend to develop a method that can quickly detect the type and content of harmful anions and metal ions in water. At present, plasma emission spectroscopy,^43^ mass spectrometry,^44^ chromatography^45^ and electrochemical analysis methods^46^ have been widely used in the detection of harmful ions in the environment. Although these detection methods can well complete the qualitative and quantitative detection of harmful ions in the environment, they have the disadvantages of relying on large-scale equipment, complex sample pretreatment, low detection efficiency, and high cost. Despite its advantages of simple operation, fast response time, low cost, naked eye identification and colorimetric detection, fluorescent probes have the disadvantage that they cannot be recovered and separated from the detection system.^47,48^ Solid-phase sensors have the advantages of convenient operation, environmental friendliness, easy separation, and reusability, and are widely used in environmental monitoring. Therefore, the development of solid-phase composite nano-fluorescence sensors has become an important direction for fluorescence sensing.^49,50^

**Scheme 1:**
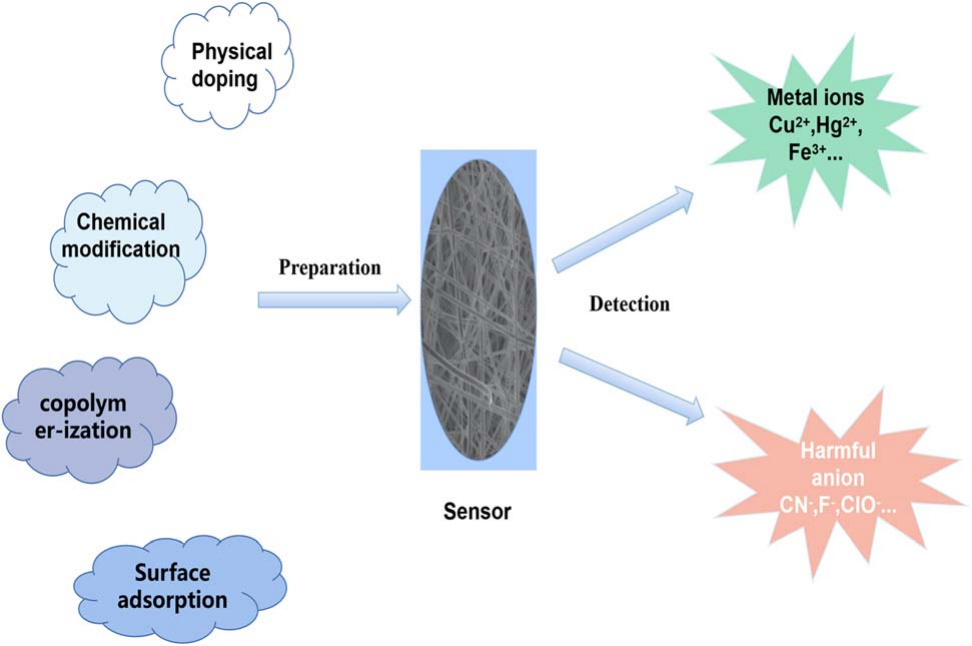
Preparation and application of electrospun fiber membrane ion sensor.

Electrospinning technology is an efficient method for the preparation of nanofibers. Compared with traditional nanofibers, electrospun nanofibers have the advantages of high porosity, ultra-high specific surface area and uniform fiber diameter, which are ideal sensing substrates. In addition, electrospinning technology is simple to operate, low cost, widely applicable (almost all linear polymers and sols) and capable of continuous production in large batches.^51,52^ Reneker once pointed out that electrospinning technology can prepare nanofibers up to one kilometer long.^53^ Yuris Dzenis define it as an effective method that can realize the preparation of “Continuous Fibers for Nanotechnology".^54^ It has been widely used in the fields of environment,^55,56^ energy,^57,58^ biomedicine,^59,60^ sensing,^61,62^ EMI shielding,^63^ fiber reinforced composites,^64,65^ smart textiles,^66^ food packaging,^67^ electrocatalysts,^68^ actuator,^69,70^ water treatment.^71–73^ Usually, some sensing units (fluorescent dyes, quantum dots, metal–organic framework luminescent metal nanoclusters, *etc.*) and nanofiber materials are combined to form a composite sensing material and prepared into test strips for sensing (Scheme 1). The current electrospun fiber test strips for colorimetric or fluorescent detection have made great progress in sensing pH,^74^ temperature,^75^ harmful ions,^76^ toxic gases^77^ and biomolecules.^78^ After the test, the test strip can be directly removed from the water, and can even be prepared as a reusable test strip. There are many ways to combine the materials of electrospun nanofibers with chemical sensor, such as physical doping, chemical modification, copolymerization, surface adsorption and self-assembly.

Physical doping method is through the sensing unit (fluorescent dye, quantum dots, metal–organic framework, *etc.*) directly dispersed in a certain concentration of polymer solution, and then using electrospinning technology to prepare nanofiber membrane. This method has the advantages of simple preparation and wide application. However, the binding mode of sensing units and polymers only relies on weak intermolecular interactions, so some hydrophilic sensing units fall off the nanofibers in aqueous environment, which affects the sensing effect. Moreover, many luminescent units will undergo aggregation-induced quenching in the aggregated state, which is not suitable for direct physical doping. The utilization rate of the large number of sensing units wrapped inside the fiber is not high.

In the copolymerization method, the sensing unit is prepared into monomer and copolymerized with some high performance polymer monomer to form block polymer, which is then prepared into nanofiber film. This method effectively avoids the shortcomings of the sensor unit falling off the fiber, but the problems of aggregation-induced quenching and the inability to utilize the sensor unit inside the fiber are not solved.

Chemical modification is made by grafting sensing units on the polymer and reusing electrospinning technology to prepare nanofibers or grafting sensing units on the surface of nanofibers. The idea is to form strong chemical bonds between the sensing unit and the polymer chain. Among them, the method of chemical modification of sensing unit on fiber surface can reduce the influence of aggregation induced quenching and make the sensing unit directly contact with the measured object, so that the sensing efficiency is higher.

The method of surface adsorption is similar to surface chemical modification, except that one uses strong chemical bond binding while surface adsorption uses electrostatic interaction binding between fiber surface and sensing unit. This method can also effectively reduce the effect of aggregation-induced quenching and enhance the utilization of sensing units.

The host-guest self-assembly method makes use of the ion–dipole, hydrogen bond, van der Waals force and hydrophobic interactions of the host and guest to form substances with specific structures. In the preparation of electrospun nanofibers, we synthesize polymers and sensing units with subject or guest structures, respectively. The modified polymer is then prepared into nanofibers, which are combined by host–guest interaction. This method is similar to surface chemical modification and surface adsorption, except that we can change the structure of host and guest by changing the conditions to regulate the binding and separation between them.

This article will review the related nanofiber sensors in the past ten years (2012–2022) from the aspects of fiber material, preparation method, sensing mechanism, and sensor performance based on the different types of ions to be detected.

## Electrospun nanofiber fluorescent sensor for detection of harmful metal ions

Generally, transition metal ions have empty orbitals and usually have strong coordination. Based on this characteristic, many compound of ligands with N, S and O atoms can be designed to detect metal ions such as organic molecular probes, surface-modified noble metal clusters and quantum dots. In order to enhance the convenience of use and sensing sensitivity of the sensor and reduce the pollution to the environment, metal-ion composite sensors based on electrospun nanofibers have made remarkable progress in the past decade. Some electrospun fiber membrane metal ion sensor base on organic molecular probes, metal nanoclusters, nanoparticles and quantum dots have been prepared by physical doping, chemical modification, copolymerization and self-assembly.

### Electrospun nanofiber fluorescent sensor for detection of Cu^2+^

#### Physical doping method

Some organic molecular and nanoparticle probes that respond to Cu^2+^ can be combined into nanofibers by physical doping to prepare nanofiber membrane sensors for detecting Cu^2+^, due to the strong interaction between sensing molecules and polymers.

**Fig. 1:**
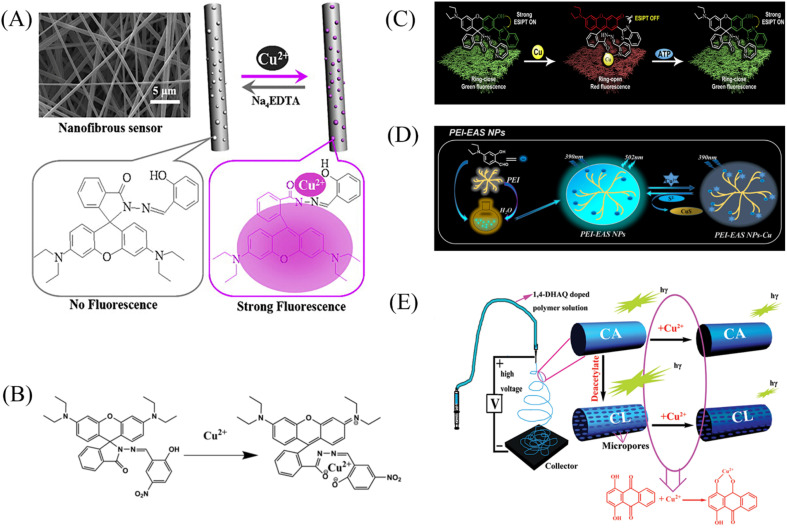
Preparation of copper ion sensor by physical doping method and their sensing mechanism. (A) Reprinted with permission from ref. 79. Copyright 2013, Elsevier. (B) Reprinted with permission from ref. 80. Copyright 2021, The Korean Fiber Society. (C) Reprinted with permission from ref. 81. Copyright 2020, Elsevier. (D) Reprinted with permission from ref. 84. Copyright 2021, Elsevier. (E) Reprinted with permission from ref. 82. Copyright 2012, *American Chemical Society*.

**Fig. 2:**
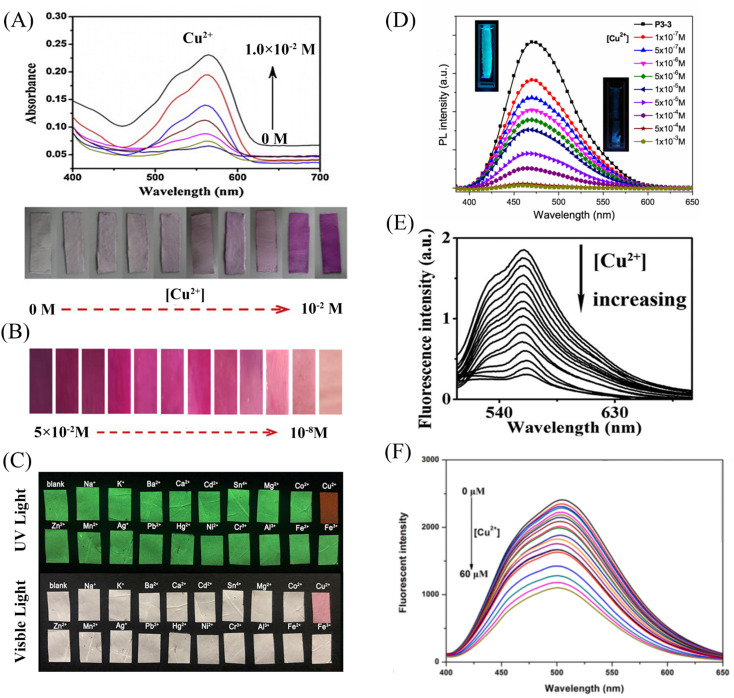
Sensing properties of copper ion sensors preparted by physical doping. (A) Reprinted with permission from ref. 79. Copyright 2013, Elsevier. (B) Reprinted with permission from ref. 80. Copyright 2021, the Korean Fiber Society. (C) Reprinted with permission from ref. 81. Copyright 2020, Elsevier. (D) Reprinted with permission from ref. 83. Copyright 2015, Springer Science Business Media New York. (E) Reprinted with permission from ref. 82. Copyright 2012, *American Chemical Society*. (F) Reprinted with permission from ref. 84. Copyright 2021, Elsevier.

Min *et al.*^79^ doped salicylaldehyde-modified rhodamine dye into poly(ether-sulfone) (PES) to prepare a sensor for copper ion response in aqueous medium by electrospinning technology. The nanofiber membrane sensor can achieve high sensitivity and selective response to copper ions. The detection limit is as low as 1.1 nM and it exhibits remarkable fluorescence enhancement and colorimetric effect. The fiber membrane shows good reusability after treatment with EDTA (Fig. 1(A) and 2(A)). Zhang *et al.*^80^ fabricated an electrospun fiber membrane from chitosan/polyacrylonitrile (CS/PAN) doped with rhodamine hydrazide salicylaldehyde Schiff base as a Cu^2+^ nanofiber membrane (NFM) sensor. The response mechanism of the NFM to copper ions is the coordination of copper ions and oxygen leads to the opening of the cyclic lactam, which causes the color change. The naked-eye detection limit of NFM is as low as 10^−8^ M, which is about three orders of magnitude lower than that of pure probe solution. The NFM shows good reusability after treatment with EDTA (Fig. 1(B) and 2(B)). In 2020, Jin *et al.*^81^ doped rhodamine-phenothiazine-based Schiff base derivative RB into polymethyl methacrylate (PMMA) to prepare a reusable ratio fluorescence-colorimetric nanofibrous membrane sensor by electrospinning technology. The excited-state intramolecular proton transfer (ESIPT) process of the probe molecule was inhibited by the coordination of copper ion with the oxygen atom on the carbonyl group on RB and the nitrogen atom on the pyridine, which manifested as a red shift of the emission peak and enhanced UV absorption and the limit of detection (LOD) is 0.11 μM/0.28 μM. After adding adenosine triphosphate (ATP), the copper ions will fall off from the RB molecule, and the ESIPT effect of the molecule will restore the blue-shift of the emission peak to the original fluorescence and weakened ultraviolet absorption (Fig. 1(C) and 2(C)). It can be seen that the Schiff base derivative based on rhodamine is a good copper ion recognition unit and the sensing mechanism of copper ion is the same. Moreover, the fiber membrane sensor prepared by physical doping method still shows good fluorescence and colorimetric detection effects, which indicates that rhodamine derivatives are not easily affected by aggregation-induced quenching. It showed good reusability after strong ligand treatment. It provides a good model for fabricating fiber membrane copper ion sensor easily.

Some phenols, pyridines and salicylaldehyde-based Schiff base derivatives can also be used as recognition probes for copper ions. Wang *et al.*^82^ prepared 1,4-dihydroxyanthraquinone (1,4-DHAQ) and cellulose acetate (CA) co-doped nanofibrous membranes (1,4-DHAQ@CA) by electrospinning technology. 1,4-DHAQ@CA was then deacetylated to obtain a microporous fibrous membrane (1,4-DHAQ@CL). Since 1,4-DHAQ can coordinate with Cu^2+^ in aqueous solution to form phenolate, resulting in obvious fluorescence quenching. The experimental results show that the fluorescence intensity of the fiber membrane has a good linear relationship in the range of copper ion concentration of 2.5 to 37.5 × 10^−9^ M, the LOD is 3 × 10^−9^ M. The nanofiber membrane can complete the specific response to copper ions under a variety of interfering metal ions. Therefore, the 1,4-DHAQ@CL fiber membrane prepared by simple doping method and deacetylation can achieve high sensitivity and selectivity for Cu^2+^ fluorescence detection. Under the treatment of Cr^3+^, the sensor shows good reusability (Fig. 1(E) and 2(E)). Lin *et al.*^83^ doped 1,10-phenanthroline fluorescence sensor (F-phen) into poly (*N*-isopropylacrylamide-*co-N*-methylol acrylamide) (P(NIPAAm-co-NMA)). A nanofiber membrane copper ion sensor with a thermally responsive switching mechanism was fabricated by electrospinning technology. The sensor exhibits a dynamic linear relationship in the range of copper ion concentration from 1.0 × 10^−5^ to 1.0 × 10^−4^ M and exhibits significant fluorescence quenching. When the temperature exceeds lower critical solution temperature (LCST), the conformational transition of poly (*N*-isopropylacrylamide (PNIPAAm) exhibits shrinkage and hydrophobic state, which leads to the inhibition of fluorescence emission by F-phen aggregation and the fibrous membrane in the hydrophobic state is also unfavorable for binding to copper ions, so the sensor exhibits characteristics of temperature-controlled copper ion detection. This approach opens up the possibility for more temperature-controlled sensor designs (Fig. 2(D)). Jin *et al.*^84^ synthesized nanoparticles (PEI-EAS NPs) using 4(*N*,*N*-diethyl) salicylaldehyde and poly(ethylene imine) (PEI). The PEI-EAS NPs were doped into PMMA and the nanofibrous membrane sensor PEI-EAS@NF was fabricated by electrospinning. PEI-EAS@NF can respond specifically to copper ions, showing the phenomenon of green fluorescence quenching. The fluorescence can be recovered by the treatment of sulfur ions, and the repeated detection of copper ions can be realized (Fig. 1(D) and 2(F)). Rhodamine derivatives have poor photostability and the spiral rings are easy to be opened under light conditions. Rhodamine-based sensors require strict light avoidance during synthesis and storage. The Schiff base derivative sensor mentioned above not only has the same good sensing performance as rhodamine derivative, but also has better photostability, which is more suitable for the preparation of solid-phase nanofiber membrane sensor.

#### Chemical modification method

Some copper ion recognition molecules can also be grafted on the surface of nanofibers by chemical modification methods. Due to the high specific surface area and porosity of electrospun nanofibers, the surface-grafted sensing unit can be well combined with copper ions, and has the advantages of fast response speed and high sensitivity.

**Fig. 3:**
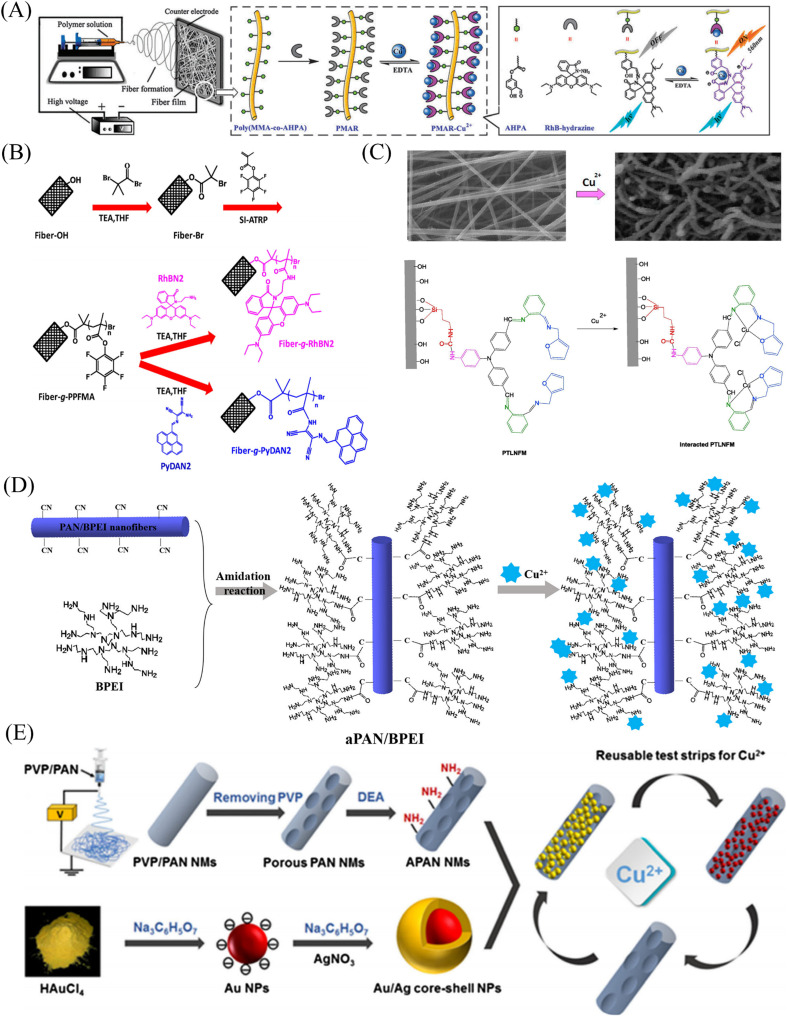
Preparation of copper ion sensor by chemical modification method and their sensing mechanism. (A) Reprinted with permission from ref. 85. Copyright 2013, *The Royal Society of Chemistry*. (B) Reprinted with permission from ref. 86. Copyright 2016, Elsevier. (C) Reprinted with permission from ref. 87. Copyright 2018, Elsevier. (D) Reprinted with permission from ref. 88. Copyright 2021, *American Chemical Society*. (E) Reprinted with permission from ref. 89. Copyright 2018, Springer-Verlag GmbH Austria, part of Springer Nature.

**Fig. 4:**
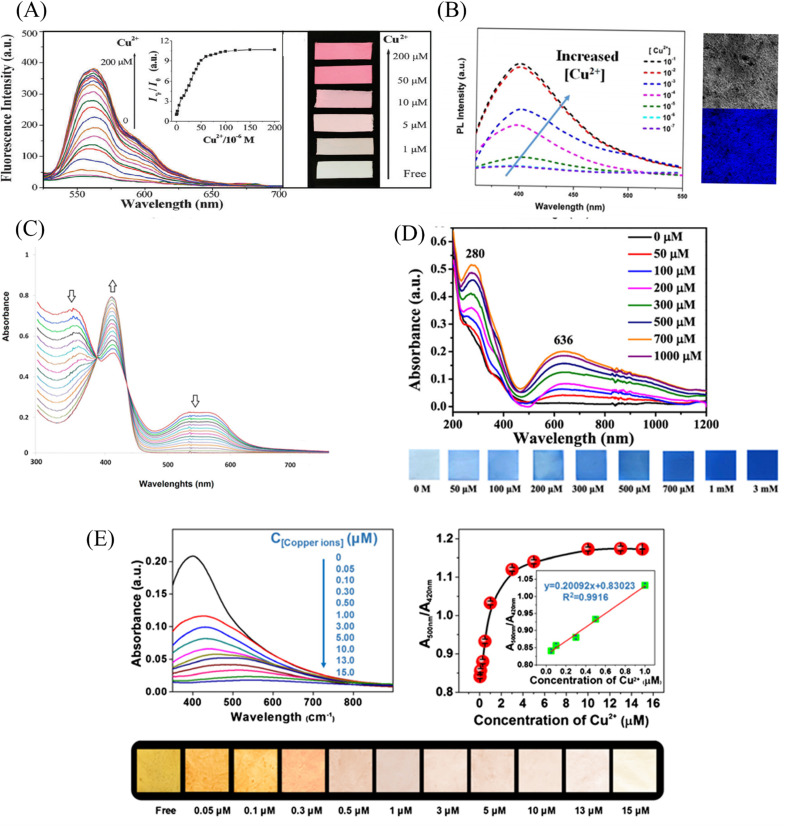
Sensing properties of copper ion sensors prepared by chemical modification. (A) Reprinted with permission from ref. 85. Copyright 2013, *The Royal Society of Chemistry*. (B) Reprinted with permission from ref. 86. Copyright 2016, Elsevier. (C) Reprinted with permission from ref. 87. Copyright 2018, Elsevier. (D) Reprinted with permission from ref. 88. Copyright 2021, *American Chemical Society*. (E) Reprinted with permission from ref. 89. Copyright 2018, Springer-Verlag GmbH Austria, part of Springer Nature.

In 2012, Wang *et al.*^85^ reported a rhodamine surface-modified poly (methyl methacrylate *co* 4-aldehyde-3-hydroxy phenyl acrylate)nanofiber membrane (PMAR), which enables real-time colorimetric-fluorescence sensing of copper ions. PMAR can sense copper ions with high efficiency and selectivity, show obvious fluorescence enhancement and colorimetric effect. Experiments show that the response time of PMAR to copper ions is very short (<10 s), with a good linear relationship in the copper ion concentration range of 1.0 × 10^−6^–2.0 × 10^−4^ M, the detection limit is 1.5 × 10^−6^ M. Copper ions can be detected repeatedly by EDTA-treated PMAR (Fig. 3(A) and 4(A)). Cho *et al.*^86^ grafted pyrene derivatives (PyDAN2) on the surface of electrospun nanofibers using poly (2-hydroxyethylmethacrylic acid-*co-N*-methacrylic acid acrylamide) (P(HEMA-*co*-NMA)) as raw material prepared a fluorescent fiber membrane sensor (Fiber-*g*-PyDAN2) with high sensitivity to copper ions. Fiber-*g*-PyDAN2 nanofibers can chelate with copper ions in aqueous solution, resulting in a blue fluorescence enhancement. The lowest and highest LOD of 10^−7^ to 10^−6^ M and 10^−2^ to 10^−1^ M, respectively. Treat it with EDTA and it can be reused at least four times (Fig. 3(B) and 4(B)). Gao *et al.*^87^ synthesized a tri-ethoxy silane-modified triphenylamine-based symmetric Schiff base derivative (L) using ultrasound-assisted technology, and grafted L onto the surface of polyvinyl alcohol (PVA) electrospun nanofiber membranes. A highly sensitive copper ion colorimetric sensor (PTLNFM) was prepared. PTLNFM can chelate Cu^2+^ in aqueous solution, resulting in changes in UV absorption. It exhibits a good linear relationship in the copper ion concentration range of 9.34 × 10^−8^ to 1.15 × 10^−5^ M and the detection limit is 1.27 × 10^−8^ M to achieve an ultrasensitive response to copper ions (Fig. 3(C) and 4(C)).

PEI is rich in amino groups and can form tetra-amino-Cu^2+^ complexes with copper ions, showing a special dark blue color, which can realize copper ion adsorption and colorimetric detection. In 2021, Shao *et al.*^88^ grafted branched polyethyleneimine (BPEI) onto the surface of polyacrylonitrile (PAN)-based electrospun nanofibrous membranes to prepare copper ion colorimetrically responsive nanofibrous membrane sensor (aPAN/BPEI NMs). The aPAN/BPEI NMs were able to adsorb copper ions in water, and the color of the fiber membrane changed from yellow to blue. With the addition of copper ions, the UV absorption peaks at 280 nm and 636 nm gradually increased, and showed a good linear relationship in the range of copper ion concentration from 0 to 700 μM. The detection limits of Cu^2+^ is *λ*_280 nm_ = 11.5 μM, *λ*_636 nm_ = 4.8 μM. And it also showed a remarkable adsorption effect on copper ions, and the adsorption capacity reached 209.53 mg g^−1^ (Fig. 3(D) and 4(D)).

Noble metal nanoparticles can be used for sensor design due to their easy surface modification and good stability. Abedalwafa *et al.*^89^ modified Au/Ag NPs with shell–core structure onto porous ammoniated PAN electrospun fibers, and successfully prepared a fiber membrane sensor Au/Ag NPs@aPAN for colorimetric detection of copper ions. With the increase of copper ions, the yellow color of the fiber membrane gradually faded. The mechanism of discoloration is the leaching of Au/Ag NPs from NFM in the presence of ammonium chloride, thiosulfate and Cu^2+^, forming a soluble complex of Ag^+^/Au^3+^/Cu^2+^-thiosulfate on their surface. The discolored fiber membrane can be restored to its original yellow color by placing it in the Au/Ag NP solution, that is, it can be regenerated. The detection limit of Au/Ag NPs@aPAN for copper ions is 50 nM (Fig. 3(E) and 4(E)).

Grafting on the fiber surface with rhodamine derivatives, Schiff base compounds, gold nanoparticles is a good way to prepare nanofiber membrane sensors that respond to copper ions. This method allows the sensing unit to be distributed on the surface of the fiber, and the detected object can fully contact the identification site. In addition, for some fluorescent dyes that are induced by aggregation, a small amount of grafting on the surface can also slow down the quenching phenomenon.

#### Copolymerization method

**Fig. 5:**
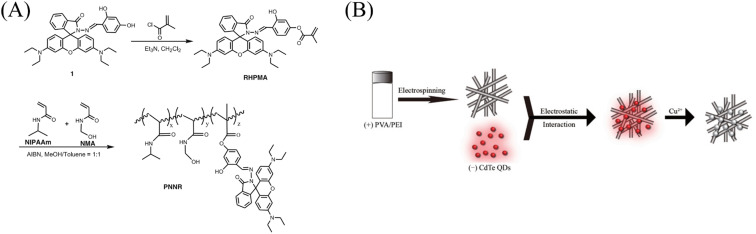
Preparation of copper ion sensor by copolymerization and surface adsorption and their sensing mechanism. (A) Reprinted with permission from ref. 90. Copyright 2016, Springer Science Business Media Dordrecht. (B) Reprinted with permission from ref. 91. Copyright 2021 Changchun Institute of Applied Chemistry, Chinese Academy of Sciences. Published by Elsevier.

**Fig. 6:**
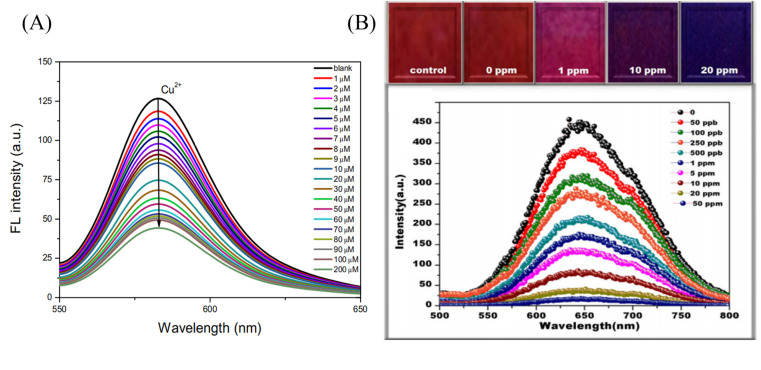
Sensing properties of copper ion sensors preparted by copolymerization and surface adsorption. (A) Reprinted with permission from ref. 90. Copyright 2016, Springer Science Business Media Dordrecht. (B) Reprinted with permission from ref. 92. Copyright 2015, Senthamizhan, A. *et al.*

Wu *et al.*^90^ firstly copolymerized thermally responsive *N*-isopropylacrylamide (NIPAAm), chemically cross-linked *N*-methylol acrylamide (NMA) and copper ion-responsive rhodamine derivative (RHPMA) to obtain polymer (PNNR). Then, a nanofiber membrane sensor (PNNR2@NFs) with temperature-controlled on and off and copper ion response was fabricated by electrospinning technology. The fluorescence intensity of PNNR2@NFs gradually weakened with the addition of copper ions, showing a good linear relationship in the concentration range of copper ions from 1 to 10 μM. The change of temperature leads to the hydrophilic–hydrophobic transition of the nanowire fibers, which shows the characteristics of temperature on and off. The sensor can be reused under the action of EDTA (Fig. 5(A) and 6(A)).

It is also an effective method to prepare the electrospun nanofiber membrane sensor. Moreover, this method makes the copper ion probe less susceptible to detachment from the fiber due to solubility.

#### Surface adsorption

Quantum dots (QDs) can be used to design chemical sensors due to their high fluorescence quantum yield and photostability. Li *et al.*^91^ used electrostatic interaction to assemble negatively charged mercaptopropionic acid (MPA)-coated CdTe QDs with surface positively charged polyethyleneimine (PEI)/polyvinyl alcohol (PVA) electrospun nanofibers to prepare a solid-state fluorescent sensor that can respond to copper ions. The fluorescence quenching degree of CdTe QDs-electrospun nanofiber assemblies increased, with the increase of Cu^2+^ concentration, and the fluorescence intensity at 652 nm showed a good linear relationship with the Cu^2+^ concentration in the range of 0.08–800 μmol L^−1^. The detection limit was 11.1 nM (S/N = 3) (Fig. 5(B)). Senthamizhan *et al.*^92^ prepared a copper ion sensor by adsorbing dithiothreitol (DTT)-coated gold nanoclusters (DTT.AuNC) onto the surface of porous cellulose acetate fibers (pCAF). With the increase of copper ions, the color of the fiber membrane gradually changed from red to blue under ultraviolet light, showing the quenching of red fluorescence, the visual detectable limit of 1 ppm (16 μM). This is due to the coordination of sulfhydryl groups on DTT surface-modified with gold nanoclusters to copper ions (Fig. 6(B)).

Using electrostatic interactions, van der Waals forces, and coordination to adsorb sensors onto nanofiber membrane surfaces is also a method to fabricate probe/nanofiber membrane composite sensors. This method is easier to operate, and the surface-adsorbed probe can quickly contact with copper ions in water, enhancing the sensing efficiency.

### Electrospun nanofiber fluorescent sensor for detection of Fe^3+^

As a transition metal, iron has abundant empty orbitals and is easy to form complexes with many ligands. Therefore, the related iron ion sensor is prepared according to this characteristic.

#### Physical doping method

**Fig. 7:**
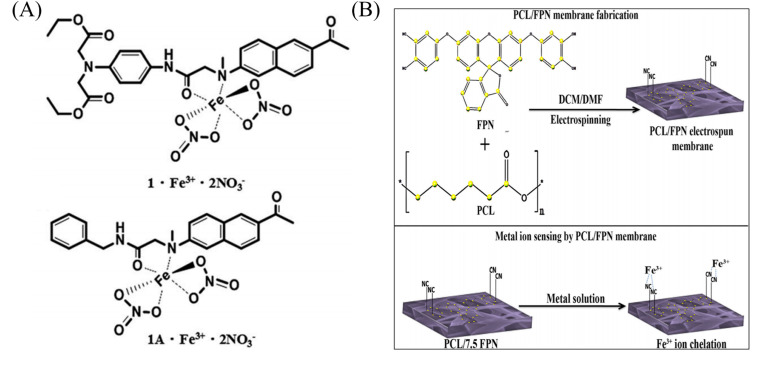
Preparation of copper ion sensor by physical doping method and their sensing mechanism. (A) Reprinted with permission from ref. 94. Copyright 2016, *The Royal Society of Chemistry*. (B) Reprinted with permission from ref. 95. Copyright 2019, Springer Science Business Media, LLC, part of Springer Nature.

**Fig. 8:**
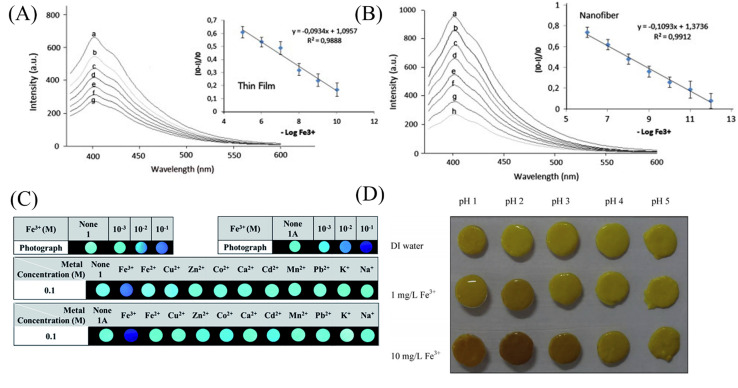
Sensing properties of iron ion sensors preparted by physical doping method. (A) and (B) Reprinted with permission from ref. 93. Copyright 2013, Elsevier. (C) Reprinted with permission from ref. 94. Copyright 2016, *The Royal Society of Chemistry*. (D) Reprinted with permission from ref. 101. Copyright 2014, Elsevier.

Some organic small molecules have more lone electron pairs, which can form chelates with paramagnetic iron ions, resulting in changes in fluorescence or color. The selective detection of iron ions can be achieved. Kacmaz *et al.*^93^ synthesized an iron-responsive fluorescent dye *N*-(4-cyanobenzylidene) isonicotinohydrazide (CBINH). By co-doping CBINH with modified ethylcellulose (EC), a nanofibrous membrane sensor CBINH@EC NFs with ultrasensitive response to iron ions was fabricated by electrospinning. CBINH@EC NFs exhibit an ultrasensitive response to Fe^3+^, in the iron ion concentration range of 10^−12^ to 10^−6^ M exhibited good linearity with detection limits as low as 0.07 fM (7 × 10^−14^ M). Under the treatment of acetate buffer, CBINH@EC NFs can achieve reversible detection of iron ions (Fig. 8(A) and (B)). Mun *et al.*^94^ synthesized naphthalene-based probe molecules 1 and 1A, and doped 1 and 1A into PMMA in DMF solution, respectively, followed by electrospinning to prepare two electrospun fiber membrane sensors NF-1 and NF-1A that could respond to iron ions. The LOD was found at 174 ppb for NF-1 and 59 ppb for NF-1A. The iron ion caused fluorescence quenching through 1 : 1 coordination with the N atom of the naphthalene ring and the carbonyl oxygen of the amide moiety (Fig. 7(A) and 8(C)). Rijin *et al.*^95^ doped 4,4′-fluoresceinoxybisphthalonitrile (FPN) into polycaprolactone (PCL) solution. A nanofibrous membrane sensor (PCL/FPN) capable of selectively detecting iron ions in aqueous solution was fabricated by electrospinning technology. The nitrogen atoms on the four cyano groups in the FPN molecule can chelate with two iron ions through weak van der Waals forces, resulting in fluorescence quenching. The response of PCL/FPN to Fe ions was tested in the concentration range of Fe ions from 10 to 70 nM, and the results showed that the detection limit of Fe ions was 2.9413 nM (Fig. 7(B)).

The complexes of rare earth elements usually have good luminescent properties. Lanthanide rare earth MOF materials have the characteristics of large Stokes shift and narrow emission wavelength, which can be used for ion detection.^96^ Zhou *et al.*^97^ prepared an Eu-MOF material using Eu^3+^ of the lanthanide series. The Eu-MOF@PAN nanofiber membrane sensor responsive to iron ions was prepared by electrospinning Eu-MOF-doped PAN solution. After adsorption of iron ions in Eu-MOF@PAN, effective collisions between Fe^3+^ and Eu^3+^ occur. This process will cause the energy of the excited state to be damaged and lead to the quenching of the fluorescence of Eu^3+^-MOF. The sensor exhibits excellent selectivity and sensitivity for iron ions with a detection limit of 63 nM. Bai *et al.*^98^ doped a chain complex (1-Eu^3+^) formed by Eu^3+^ and organic molecular ligands into PAN solution, and fabricated an iron ion-responsive nanofibrous membrane sensor (1-Eu^3+^@PAN). The sensor showed obvious red fluorescence quenching under iron ion treatment, and the detection limit was 6.685 × 10^−4^ M.

As an environmentally friendly material, biomaterials have good application prospects and can be used in the fields of environment, energy and sensing.^99,100^ Saithongdee *et al.*^101^ prepared a nanofibrous membrane sensor from a curcumin-doped zein solution by electrospinning and amidation cross-linking under the conditions of citric acid and heating. The sensor is able to achieve a colorimetric response to iron ions with a color change from yellow to brown with an optical detection limit of 0.4 mg L^−1^ (Fig. 8(D)).

Many probe molecules, MOFs, and biological matrices *etc.* Which can selectively detect iron ions can be mixed with some polymers by means of physical doping to prepare nanofiber membrane sensors that respond to iron ions by electrospinning. This simple mixing method is suitable for a wide range of linear polymers with a wide range of applications and is easy to operate. However, due to simple doping, there is no strong connection between the sensing unit and the polymer, and the polymer and the sensing unit are easily separated by solvents. For some sensing units that are easily soluble in water, they are easy to dissolve in water under the water system, which affects the sensing effect.

#### Chemical modification method

Direct chemical modification of sensing molecules onto polymers and subsequent fabrication into nanofibrous membranes can also be used for the detection of iron ions.

**Fig. 9:**
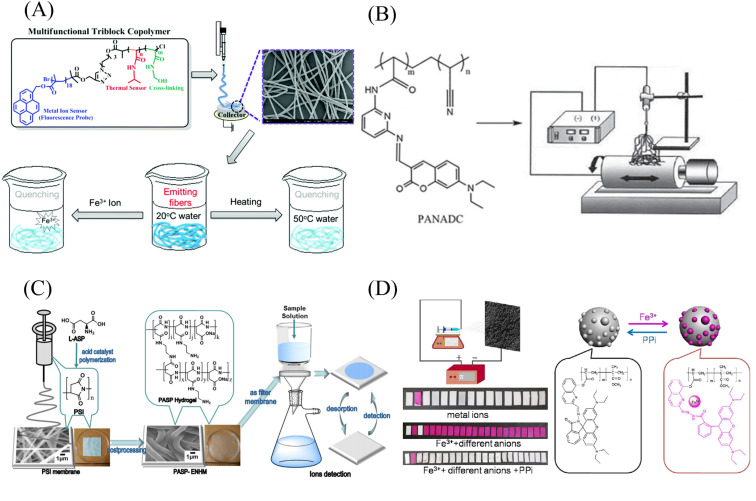
Preparation and sensing mechanism of iron ion sensor by chemical modification and copolymerization. (A) Reprinted with permission from ref. 102. Copyright 2015, *The Royal Society of Chemistry*. (B) Reprinted with permission from ref. 103. Copyright 2018, *Journal of The Brazilian Chemical Society*. (C) Reprinted with permission from ref. 104. Copyright 2019, American Chemical Society. (D) Reprinted with permission from ref. 105. Copyright 2015, Elsevier.

**Fig. 10:**
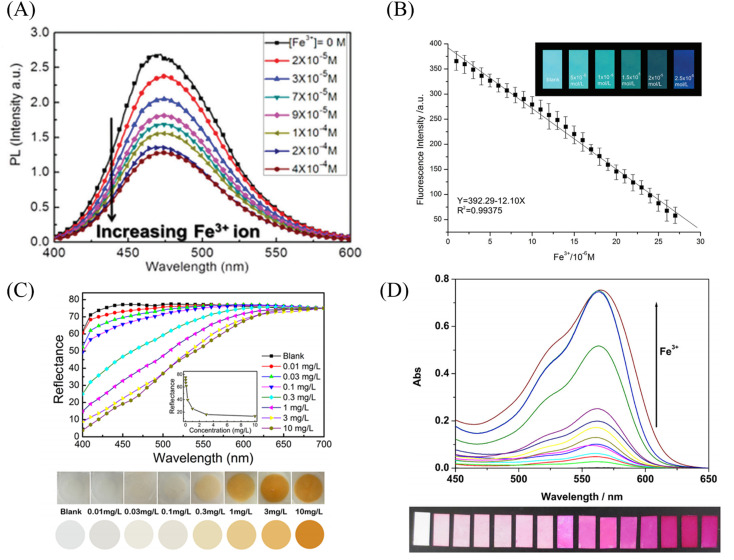
Sensing properties of iron ion sensors preparted by chemical modification and copolymerization. (A) Reprinted with permission from ref. 102. Copyright 2015, *The Royal Society of Chemistry*. (B) Reprinted with permission from ref. 103. Copyright 2018, *Journal of The Brazilian Chemical Society*. (C) Reprinted with permission from ref. 104. Copyright 2019, *American Chemical Society*. (D) Reprinted with permission from ref. 105. Copyright 2015, Elsevier.

Wang *et al.*^102^ used chemical modification to graft pyrene derivatives to triblock copolymers to form a pyrene-containing copolymer (PPy-*b*-PNIPAAm-*b*-PNMA). The pyrene-containing copolymers were then prepared into nanofibrous membranes by electrospinning. The fibers are aggregated from nanospheres formed by self-assembly of PPy-*b*-PNIPAAm-*b*-PNMA copolymer, and the pyrene-containing molecules are on the outermost side of the nanospheres. The fiber membrane can quench the fluorescence in response to iron ions and temperature. Even at the concentration of 10^−5^ M iron ion, the fluorescence of the fiber membrane can be quenched (Fig. 9(A) and 10(A)). Zhou *et al.*^103^ modified coumarin derivatives on copolymers of acrylic acid and acrylonitrile (PANA) to prepare iron ion-responsive nanofibrous membranes (PANADC) by electrospinning. The fiber membrane can be used for the adsorption and detection of iron ions, and the fluorescence is quenched with the addition of iron ions to the fiber membrane, and the detection limit and adsorption amount are 10.63 μM and 13.93 mg g^−1^, respectively (Fig. 9(B) and 10(B)).

Utilizing the adsorption of some amino acids to iron ions can also be used to detect iron ions by chemical cross-linking after some amino acids are prepared into polymers. Zhang *et al.*^104^ prepared poly(aspartic acid) (PASP) into nanofibrous membranes by electrospinning and then cross-linked with ethylenediamine to obtain an electrospun fibrous hydrogel film (PASP-ENHM). PASP-ENHM showed a colorimetric change from white to yellow under iron treatment with a detection limit of 0.1 mg L^−1^. And the fiber membrane can continue to be used for the detection of iron ions after being treated with EDTA (Fig. 9(C) and 10(C)).

### Copolymerization method

Directly polymerizing molecular probes with other monomers is also a method for preparing sensing materials. Li *et al.*^105^ synthesized a rhodamine Schiff base derivative monomer RQ, RQ and methyl acrylate were prepared by emulsion polymerization to obtain a block copolymer poly(MMA-*co*-RQ). Poly(MMA-*co*-RQ) was obtained by electrospinning prepared nano-films stacked with nano-microspheres. The film can be used for rapid colorimetric detection of iron ions from white to pink with a detection limit of 1.19 μM, and can be reused after treatment with ppi solution (Fig. 9(D) and 10(D)).

The iron ion sensor prepared by the above chemical modification method has good sensing performance. And it solves the problem that the sensing unit is easy to fall off in the physical co-doping method.

### Electrospun nanofiber fluorescent sensor for detection of Hg^2+^

Gold nanoparticles, rhodamine derivatives and quantum dots can form composite fiber membrane sensors that respond to mercury ions through physical doping, chemical modification, surface adsorption, self-assembly, and copolymerization.

#### Physical doping method

Senthamizhan *et al.*^106^ prepared a nanofibrous membrane responsive to mercury ions by doping gold nanoclusters (AuCN) into polyvinyl alcohol solution. The fiber membrane showed significant fluorescence quenching effect on mercury ions with a detection limit of 1 ppb (Fig. 12(A)).

**Fig. 11:**
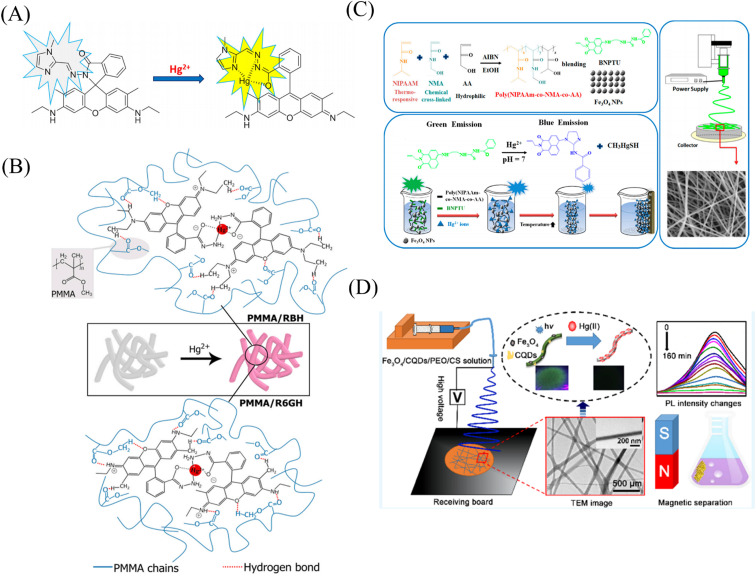
Preparation and sensing mechanism of mercury ions sensor by physical doping (A) reprinted with permission from ref. 107. Copyright 2017, Elsevier. (B) Reprinted with permission from ref. 108. Copyright 2021, Elsevier. (C) Reprinted with permission from ref. 109. Copyright 2017, MDPI. (D) Reprinted with permission from ref. 110. Copyright 2018, *American Chemical Society*.

**Fig. 12:**
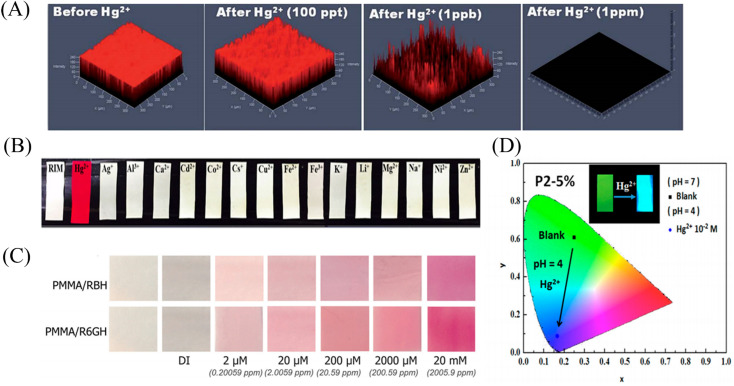
Sensing properties of mercury ion sensors prepared by physical doping (A) reprinted with permission from ref. 106. Copyright 2015, *The Royal Society of Chemistry*. (B) Reprinted with permission from ref. 107. Copyright 2017, Elsevier. (C) Reprinted with permission from ref. 108. Copyright 2021, Elsevier. (D) Reprinted with permission from ref. 109. Copyright 2017, MDPI.

Rao *et al.*^107^ synthesized a mercury ion chemical sensor RIM using rhodamine 6G as a raw material. The RIM and polyurethane were mixed uniformly, and a nanofiber membrane was prepared by electrospinning. The fibrous membrane is able to respond to mercury ion, the mechanism of sensing of mercury ion by RIM is the ring opening of spirolactum resulting in enhanced fluorescence (Fig. 11(A) and 12(B)). Girdthep *et al.*^108^ doped rhodamine B hydrazide (RBH) and rhodamine 6G hydrazide (R6GH) into PMMA, respectively. And prepared PMMA/RBH and PMMA/R6G mercury ion colorimetric and fluorescent nanofiber membrane sensor by electrospinning. Both sensors can chelate with mercury ions in a 2 : 1 ratio, resulting in a white-to-pink color change and fluorescence enhancement due to the opening of the spiro ring. The detection limits of the PMMA/RBH and PMMA/R6G are 0.8–1.099 ppb and 3.6–4.3 ppb, respectively. The two sensors can be recycled under the treatment of EDA solution (Fig. 11(B) and 12(C)).

Liang *et al.*^109^ fabricated a temperature-on–off magnetic nanofiber membrane sensor (P2-5%ESNFs) for mercury ion response. The sensor was prepared by electrospinning by mixing poly (NIPAAm-*co*-NMA-*co*-AA) (P2), naphthalimide derivative (BNPTU) and Fe_3_O_4_ nanoparticles. P2-5% ESNFs showed the performance of temperature control in response to mercury ions. The temperature change changed the hydrophilic–hydrophobic state of the fiber membrane, and the chelation of mercury ions and BNPTU was more likely to occur in the hydrophilic state of the fiber membrane. Therefore, the on and off effects of temperature are exhibited. The response of P2-5% ESNFs to mercury ions changed from green to blue. The addition of Fe_3_O_4_ makes the fiber membrane magnetic for easy removal from water (Fig. 11(C) and 12(D)). Li *et al.*^110^ prepared a one-dimensional electrospun fiber membrane material using a mixed solution of polyethylene oxide (PEO), chitosan (CS), Fe_3_O_4_ and CQDs. The fiber membrane material can monitor the adsorption of mercury ions by real-time fluorescence monitoring. The adsorption of mercury ions reaches equilibrium within 100 min, and the maximum single-layer adsorption capacity is 148.148 mg g^−1^. The fluorescence of the fiber membrane was gradually quenched with the adsorption of mercury ions. The fiber membrane is magnetic and can be easily removed from the aqueous solution, due to the addition of Fe_3_O_4_ (Fig. 11(D)). Mercury ion probes based on rhodamine derivatives, naphthalimide derivatives, quantum dots and gold nanoparticles can be simply doped into polymers to quickly prepare electrospun fiber membrane sensors with good sensing effects. However, the sensing unit inside the fiber in this preparation method is not used, and the sensing unit on the surface of the fiber is easy to fall off due to the combination of the polymer only by weak molecular force, which affects the sensing effect.

#### Chemical modification method

**Fig. 13:**
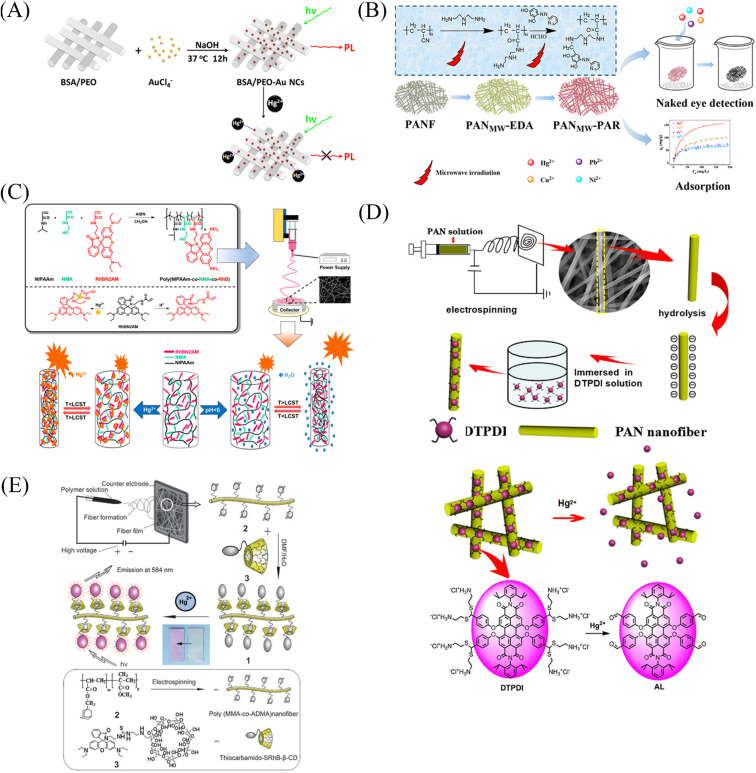
Preparation and sensing mechanism of mercury ions sensor by chemical modification, self-assembly and surface adsorption (A) reprinted with permission from ref. 111. Copyright 2012, Elsevier. (B) Reprinted with permission from ref. 112. Copyright 2019, *American Chemical Society*. (C) Reprinted with permission from ref. 113. Copyright 2018, MDPI. (D) Reprinted with permission from ref. 114. Copyright 2016, Elsevier. (E) Reprinted with permission from ref. 115. Copyright 2012, *The Royal Society of Chemistry*.

**Fig. 14:**
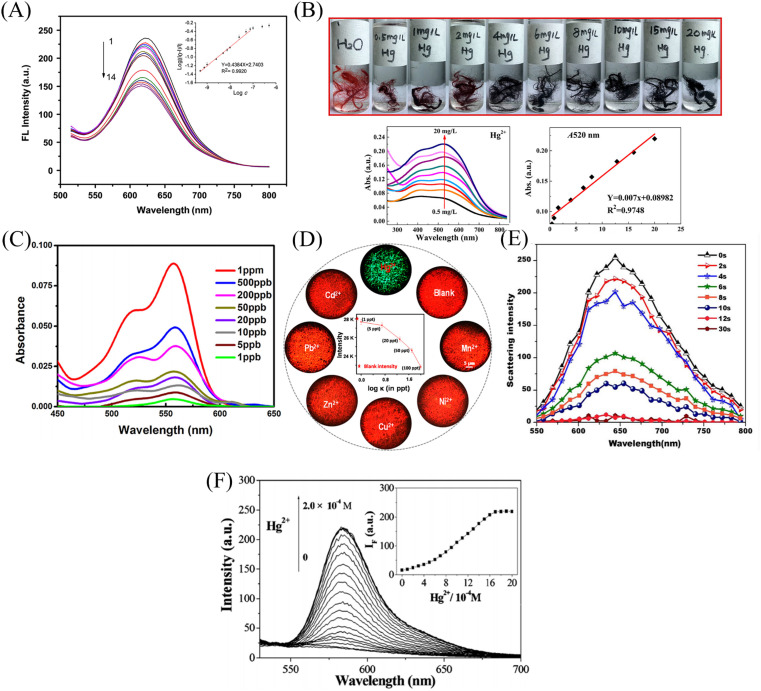
Sensing properties of mercury ion sensors prepared by chemical modification, self-assembly and surface adsorption (A) reprinted with permission from ref. 111. Copyright 2012, Elsevier. (B) Reprinted with permission from ref. 112. Copyright 2019, *American Chemical Society*. (C) Reprinted with permission from ref. 114. Copyright 2016, Elsevier. (D) Reprinted with permission from ref. 115. Copyright 2014, *American Chemical Society*. (E) Reprinted with permission from ref. 116. Copyright 2015, Anitha Senthamizhan *et al.* (F) Reprinted with permission from ref. 117. Copyright 2012, *The Royal Society of Chemistry*.

Cho *et al.*^86^ fabricated a mercury ion sensor (Fiber-*g*-RhBN2) by modifying a rhodamine B derivative (RhBN2) on the surface of poly (HEMA-*co*-NMA) electrospun nanofibers. With the addition of mercury ions, the fluorescence intensity of Fiber-*g*-RhBN2 at 580 nm gradually increased. The lowest and highest detection limits were 10^−5^ to 10^−6^ M and 10^−2^ to 10^−1^ M, respectively. Furthermore, treatment of Fiber-*g*-RhBN2 with EDTA enables the reuse of the sensor. Cai *et al.*^111^ reduced HAuCl_4_ to Au-NCs with BSA by *in situ* preparation on bovine serum albumin/polyethylene oxide (BSA/PEO) electrospun fibrous membrane and immobilized on the surface and inside of the fibrous membrane. The modified fiber membranes (BSA/PEO-Au-NCs) can exhibit fluorescence quenching in the presence of mercury ions with a detection limit of 57 pM (Fig. 13(A) and 14(A)). Deng *et al.*^112^ grafted 4-(2-pyridylazo)-resorcinol (PAR) onto PAN electrospun fibers to prepare a sensor (PANMW-PAR) for colorimetric detection of mercury ions and adsorption of mercury ions. PANMW-PAR fibers have selective detection ability for Hg^2+^ with a detection limit of 35 μg L^−1^ (Fig. 13(B) and 14(B)).

#### Copolymerization method

Chen *et al.*^113^ synthesized a rhodamine derivative (RhBN2AM). Poly(*N*-isopropylacrylamide, poly(*N*-methylolacrylamide) and RhBN2AM were synthesized by free radical solution polymerization to obtain a copolymer (poly(NIPAAm-*co*-NMA-*co*-RhBN2AM)), and the copolymer was dot-spun to prepare a nanofiber membrane P3. The fibrous membrane can turn into orange fluorescence in response to mercury ions. In addition, the NIPAAm in P3 also has the function of temperature-controlling the response of mercury ions on and off. When the temperature exceeds the LCST (45 °C), the fluorescence of the fiber membrane increases instantaneously (Fig. 13(C)).

#### Surface adsorption

Ma *et al.*^114^ synthesized a dithioacetal-modified pyreneimide fluorescent sensor DTPDI. Subsequently, DTPDI was adsorbed on the surface of PAN electrospun fiber membrane by electrostatic interaction to form a fiber membrane sensor FNFM that could respond to mercury ions. FNFM produced a lipid-soluble dye AL in the presence of mercury ions, which was exfoliated from the surface of the fibrous membrane upon treatment with dichloromethane. The mercury ion content can be determined by measuring the UV absorption intensity of AL exfoliated in the solution, and the detection limit is 1 ppb (Fig. 13(D) and 14(C)). Ghosh *et al.*^115^ prepared an electrospun nanofibrous membrane by blending nylon 6 (N6) and fluorescein isothiocyanate (FITC). Then, Au@BSA nanoclusters were adsorbed by nanofiber membrane to prepare a sensor Au@BSA/FITC/N6 for the selective detection of mercury ions. Under the treatment of mercury ions, the red fluorescence was gradually quenched and returned to green fluorescence, which could also respond at a concentration of 1 ppt mercury ions (Fig. 14(D)). Senthamizhan *et al.*^116^ fabricated a Hg^2+^ sensor (AuNC*PCL-NF) by adsorbing a layer of gold nanoclusters on the surface of polycaprolactone electrospun fibers (PCL-NF). The amalgam caused the red fluorescence of the gold nanoclusters to be quenched in a very short time, and the detection limit was at the ppt level (Fig. 14(E)).

#### Self-assembly

Wang *et al.*^117^ used β-cyclodextrin-modified rhodamine derivatives (thiocarbamido-SRhB-β-CD) as the host and poly (MMA-*co*-ADMA) electrospun nanofibers as the guest. A sensor for colorimetric and fluorescence detection of mercury ions was prepared by host–guest self-assembly. With the addition of mercury ions, the sensor exhibited an orange fluorescence enhancement, and the color changed from white to pink under visible light. The detection limit was 6.0 × 10^−5^ mol L^−1^ (Fig. 13(E) and 14(F)).

Nanofiber membrane sensors were prepared by copolymerization to form block polymers between mercury ion probes and polymers. It can effectively prevent the probe from wandering into the detection environment, but the probe utilization rate and physical doping method are not much different. Chemical modification, surface adsorption and self-assembly methods can modify the mercury ion sensing unit on the surface of the fiber membrane to increase the utilization rate of the mercury ion sensing unit and reduce the aggregation and quenching phenomenon of fluorescent molecules.

### Electrospun nanofiber fluorescent sensor for detection of Pb^2+^

Some biological matrix materials and noble metal nanoclusters are very useful for preparing lead ion nanofiber membrane sensors by co-doping, chemical modification and other methods.

#### Physical doping method

**Fig. 15:**
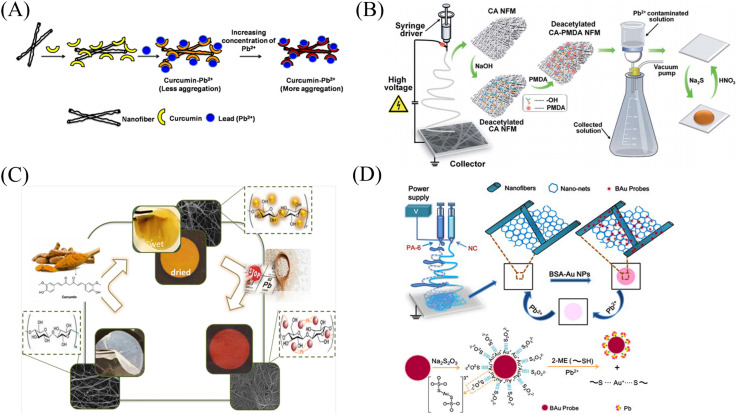
Preparation and sensing mechanism of Pb^2+^ sensor. (A) Reprinted with permission from ref. 118. Copyright 2015, Elsevier. (B) Reprinted with permission from ref. 119. Copyright 2015, *The Royal Society of Chemistry*. (C) Reprinted with permission from ref. 120. Copyright 2021, Iranian Chemical Society. (D) Reprinted with permission from ref. 121. Copyright 2013, Elsevier.

**Fig. 16:**
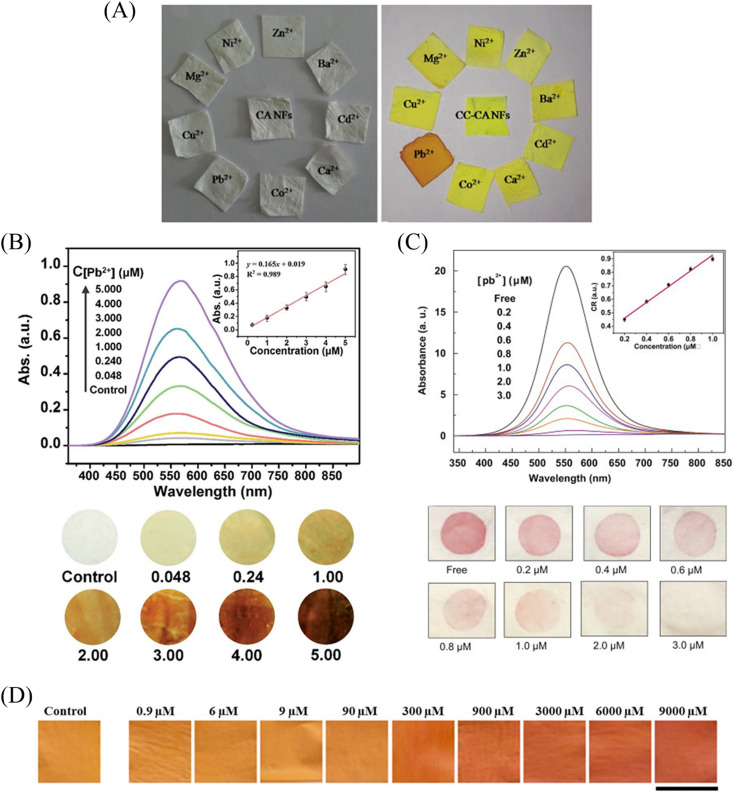
Sensing properties of Pb^2+^ sensors. (A) Reprinted with permission from ref. 118. Copyright 2015, Elsevier. (B) Reprinted with permission from ref. 119. Copyright 2015, *The Royal Society of Chemistry*. (C) Reprinted with permission from ref. 121. Copyright 2021, Iranian Chemical Society. (D) Reprinted with permission from ref. 120. Copyright 2013, Elsevier.

Raj *et al.*^118^ prepared a fiber membrane sensor CC-CA for colorimetric detection of lead ions by electrospinning a mixed solution of curcumin and cellulose acetate (CA). CC-CA changed from yellow to orange under the treatment of lead ions with a detection limit of 20 μM (Fig. 15(A) and 16(A)).

#### Chemical modification method

Li *et al.*^119^ prepared cellulose acetate (CA) into nanofibrous membranes by electrospinning technology, and then deacetylated the CA nanofibrous membranes to obtain DCA fibrous membranes. A sensor DCA-PMAD for colorimetric detection of lead ions can be obtained by grafting pyromellitic anhydride (PMAD) onto the surface of DCA fiber membrane. With the addition of lead ions, DCA-PMAD changed from white to dark yellow–brown, and the detection limit was 0.048 mM. And DCA-PMAD also had a strong adsorption capacity for lead ions, and the adsorption capacity was 326.80 mg g^−1^. Under the treatment of EDTA, the sensor is reusable (Fig. 15(B) and 16(B)).

#### Surface adsorption method

Sheikhzadeh *et al.*^120^ prepared electrospun nanofibrous membranes (BCNFs) from bacterial cellulose. The lead ion colorimetric sensor (BCNF-CU) was then prepared by adsorbing curcumin with BCNF. The naked eye detection limit and the calculated detection limit of BCNF-CU were 9 μM and 0.9 μM, respectively. And applied to the detection of lead ions in rice samples (Fig. 15(C) and 16(D)). Li *et al.*^121^ prepared polyamide-6/nitrocellulose (PA-6/NC) electrospun fiber membranes by bicomponent alternating two-flow electrospinning technique. The bovine serum albumin-modified gold nanoparticles (BAu probe) were adsorbed onto the surface of the fiber membrane by assembly method to obtain a sensor BAu probe@PA-6/NC that can be used for the colorimetric detection of lead ions. The color of the fiber membrane changed from pink to white with increasing lead ion concentration, and the detection limit was 0.2 μM. And it can be reused after soaking with BAu probe solution (Fig. 15 (D) and 16 (C)).

### Electrospun nanofiber membranes for Al^3+^ detection

**Fig. 17:**
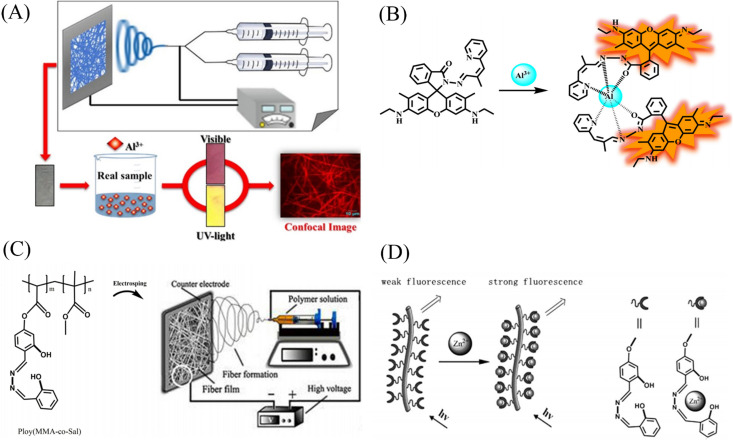
Preparation and sensing mechanism of Zn^2+^, Al^3+^ sensor. (A) and (B) Reprinted with permission from ref. 122. Copyright 2016, Elsevier. (C) and (D) Reprinted with permission from ref. 124. Copyright 2017, Chen Zhou *et al.*

**Fig. 18:**
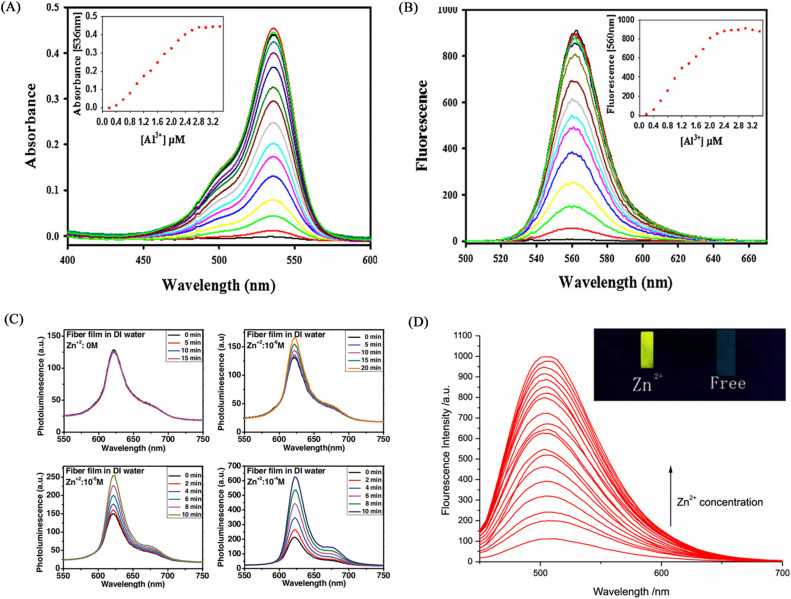
Sensing properties of Zn^2+^, Al^3+^ sensor. (A) and (B) reprinted with permission from ref. 122. Copyright 2016, Elsevier. (C) Reprinted with permission from ref. 123. Copyright 2013, Wiley-VCH (D) reprinted with permission from ref. 124. Copyright 2017, Chen Zhou *et al.*

Kim *et al.*^122^ blended a rhodamine-based colorimetric-fluorescent chemical sensor (R2PP) with polyurethane to prepare an aluminum ion-responsive fiber membrane sensor by electrospinning. Under the treatment of aluminum ions, the fibrous membrane changed from white to pink and showed a significant enhancement of yellow fluorescence, and the detection limit was 8.5 × 10^−9^ M. The sensing mechanism is due to the formation of a 2 : 1 complex between R2PP and aluminum ions, which results in the opening of the spiro ring structure of R2PP, which changes the conjugation structure and changes the UV and fluorescence. In addition, it can be reused under the treatment of EDTA (Fig. 17(A), (B) and 18(A), (B)).

### Electrospun nanofiber membranes for Zn^2+^ detection

Syu *et al.*^123^ doped the zinc ion probe meso-2,6-dichlorophenyltripyrrinone (TPN-Cl_2_) into poly(2-hydroxyethyl methacrylate) (PHEM) solution and fabricated a fiber membrane sensor by electrospinning. The sensor can respond to zinc ions in the normal physiological range, and the time resolution can reach 5 min at the zinc ion concentration of 10^−6^ M (Fig. 18(C)). Zhou *et al.*^124^ obtained poly (MMA-*co*-Sal) by polymerizing and modifying 2-hydroxy-4-acryloyloxy-benzaldehyde (HAB), methyl methacrylate (MMA) and salicylaldehyde-hydrazine as raw materials. The polymer poly (MMA-*co*-Sal) can be prepared into nanofibrous membranes by electrospinning technology. The fiber membrane can be used for fluorescence detection and adsorption of zinc ions. The fluorescence at 504 nm gradually increased with the addition of zinc ions, the detection limit was 1.95 × 10^−5^ mol L^−1^, and the adsorption capacity was 11.45 mg g^−1^ (Fig. 17(C), (D) and 18(D)).

Regardless of the form of binding, the sensing of metal ions is always inseparable from the coordination and oxidation of metal ions. Therefore, the design of metal ion sensing mainly considers the influence of the way the sensing unit is introduced on the sensor performance.

### Electrospun nanofiber fluorescent sensor for detection of harmful anions

The preparation of electrospun fiber membrane sensor for harmful anion detection can utilize physical doping, chemical grafting, and host–guest self-assembly to combine CDs, organic molecular probes, and noble metal nanoparticles with polymers.

### Electrospun nanofiber fluorescent sensor for detection of CN^−^

#### Physical doping method

**Fig. 19:**
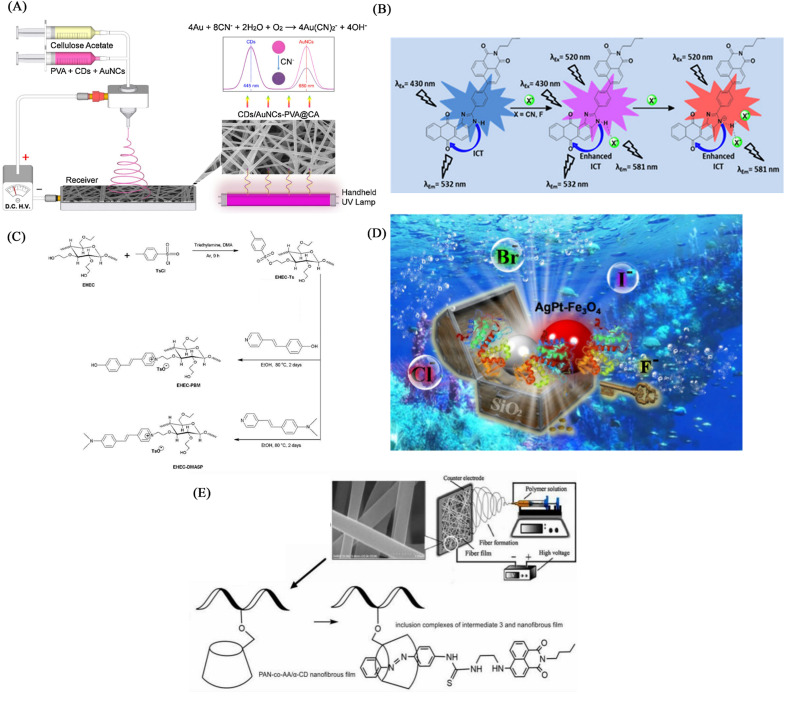
Preparation and sensing mechanism of CN^−^, F-sensor (A) reprinted with permission from ref. 125. Copyright 2019, Elsevier. (B) Reprinted with permission from ref. 126. Copyright 2018, Elsevier. (C) Reprinted with permission from ref. 127. Copyright 2020, Elsevier. (D) Reprinted with permission from ref. 128. Copyright 2022, *American Chemical Society*. (D) Reprinted with permission from ref. 129. Copyright 2021, Huan Zhang *et al.*

**Fig. 20:**
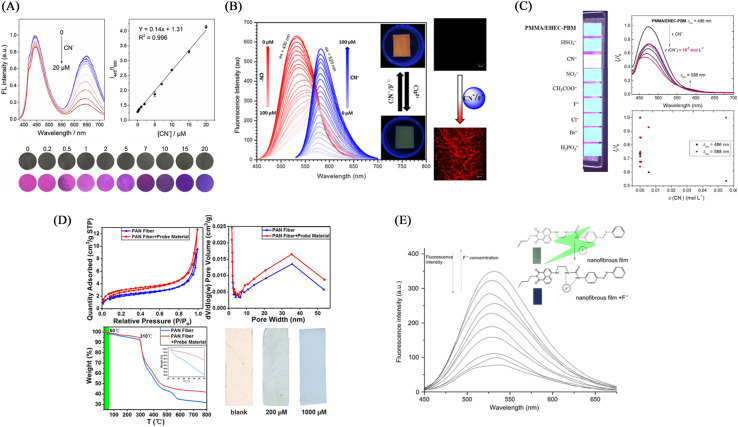
Sensing properties of CN^−^, F-sensor (A) reprinted with permission from ref. 125. Copyright 2019, Elsevier. (B) Reprinted with permission from ref. 126. Copyright 2018, Elsevier. (c) Reprinted with permission from ref. 127. Copyright 2020, Elsevier. (D) Reprinted with permission from ref. 128. Copyright 2022, *American Chemical Society*. (E) Reprinted with permission from ref. 129. Copyright 2021, Huan Zhang *et al.*

Hu *et al.*^125^ first electrospinned the DMF : acetone = 2 : 1 solution of cellulose acetate for 30 minutes, then used the PVA aqueous solution in which CDs and AuNC were uniformly dispersed for 30 minutes, and finally used the organic cellulose acetate solution. Solution electrospinning for 30 min. A fluorescent nanofiber membrane sensor (CDs/AuNCs-PVA@CA NFM) for ratiometric detection of cyanide was successfully fabricated. The CDs/AuNCs-PVA@CA NFM fluorescence intensity of 445 nm/650 nm gradually increased with the addition of cyanide, showing a ratiometric detection of cyanide. Since the relative content of Au (0) decreased due to the etching effect of cyanide on AuNCs, the fluorescence intensity at 650 nm of AuCNs decreased, while the fluorescence intensity at 445 nm of CDs was not affected, so NFM showed the effect of ratiometric detection. Experiments show that the detection limit of NFM is 0.15 μM, which is much lower than the WHO standard for cyanide content in drinking water (Fig. 19(A) and 20(A)).

Kim *et al.*^126^ synthesized an imidazole derivative (M) based on naphthalimide and anthraquinone for cyanide detection. A cyanide detection test strip was prepared by blending M and polyurethane in dimethylacetamide and electrospinning. The test strip showed the effect of colorimetric and fluorescence detection under the treatment of cyanide. It changes from green to orange under visible light, and exhibits an orange–red fluorescence enhancement under UV light irradiation. The reason for this change is that the cyanide can strongly interact with the H on the N–H unit of imidazole and dissociate the proton, resulting in an enhanced ICT effect but a sharp change in UV and fluorescence (Fig. 19(B) and 20(B)).

#### Chemical modification method

Dreyer *et al.*^127^ grafted 4-(2-(pyridin-4-yl)vinyl) phenol (PBM) onto ethyl(hydroxyethyl)cellulose (EHCE). The modified EHCE and PMMA were blended at a mass ratio of 5 : 2 (wt/wt) to obtain a cyanide fiber membrane sensor PMMA/EHEC-PBM by electrospinning. PMMA/EHEC-PBM has good hydrophilicity and can be used for the detection of enhancers in total aqueous solution. With the addition of cyanide, the fluorescence at 486 nm is gradually quenched, and the fluorescence at 588 nm is gradually enhanced. The sensing mechanism is due to the change of fluorescence caused by the proton detachment of the phenolic hydroxyl group in the presence of cyanide.^31^ The detection limit and quantification limit of this sensor were 2.15 × 10^−5^ and 7.17 × 10^−5^ mol L^−1^, respectively (Fig. 19(C) and 20(C)).

### Electrospun nanofiber fluorescent sensor for detection of F^−^

As a strong electronegative element, F^−^ easily forms a hydrogen bond with N–H or O–H to deprotonate the molecule, thereby causing the change of the conjugated structure and the change of fluorescence and color. In addition, fluoride, as a lewis acid, can react with silicon-containing compounds, and fluoride sensors can be designed according to this characteristic.

#### Physical doping method

The (M) synthesized by Kim *et al.*^126^ can also be used for the detection of fluoride compounds, and the test strip prepared by electrospinning technology can also be used for the detection of fluoride compounds from the perspective of colorimetry and fluorescence. The detection mechanism is due to the strong interaction of the fluoride ion with the H atom on the N–H leading to the dissociation of the proton. This change can enhance the ICT effect of the molecule and achieve a significant change in UV fluorescence (Fig. 19(B) and 20(B)). Qiu *et al.*^128^ fabricated AgPt–Fe_3_O_4_ nanoparticles by modifying iron tetroxide nanoparticles with Ag and Pt. Then, AgPt–Fe_3_O_4_ was wrapped with SiO_2_ to prepare AgPt–Fe_3_O_4_@SiO_2_ NPs. They were doped into the DMF solution of PAN, and then the nanofiber membrane F^−^ sensor was fabricated by electrospinning technology. In the presence of F^−^, the sensor realizes a good colorimetric effect from light pink to blue, and the limit of detection is 13.73 μM. The mechanism of this color development is due to the etching effect of F^−^ on SiO_2_, which makes the outer shell of SiO_2_ fall off and exposes the active part of AgPt–Fe_3_O_4_ inside, which oxidized and changes color. Can be used for colorimetric detection of F^−^ in total aqueous solution (Fig. 19(D) and 20(D)).

#### Self-assembly

Zhang *et al.*^129^ used a β-cyclodextrin (β-CD)-modified copolymer of acrylonitrile and acrylic acid (PAN-*co*-AA) electrospun fiber membrane (PAN-*co*-AA/β-CD) as the host, and the conjugated A nitrobenzene-modified naphthalimide derivative (intermediate 3) served as a guest. The host–guest self-assembly method was used to combine them to form a fiber membrane sensor for F-high selective detection. Due to the strong electronegativity of F^−^, it will form a hydrogen bond interaction with the N–H unit in the intermediate 3 on the surface of the fiber membrane, and make the proton on the N–H dissociate, which makes the electron transfer to the homo-orbital of the excited state conjugated structure through the PET effect, and hindering the movement of electrons reduces the fluorescence. The fiber membrane senses for F^−^ and also has good adsorption capacity for F^−^, with an adsorption capacity of 16.67 mg g^−1^ (Fig. 19(E) and 20(E)).

### Electrospun nanofiber sensor for detection of ClO^−^

**Fig. 21:**
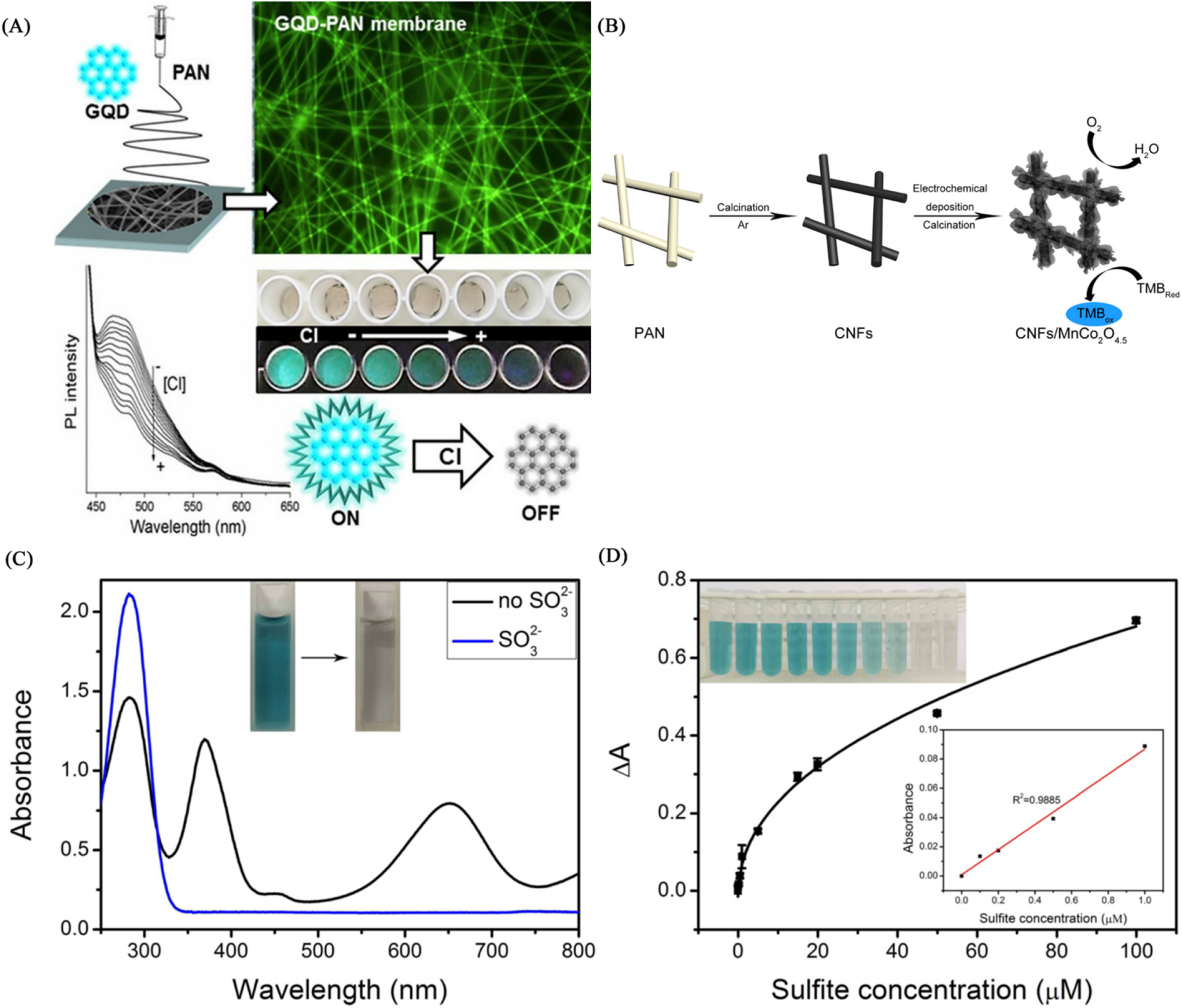
Fabrication and properties of ClO^−^, So_3_^2−^nanofiber membrane sensors (A) reprinted with permission from ref. 130. Copyright 2018, Elsevier. (B), (C) and (D) reprinted with permission from ref. 131. Copyright 2017, IOP Publishing Ltd.

Virginia *et al.*^130^ used electrospinning technology to encapsulate active chlorine-responsive graphene quantum dots (GQDs) in PAN to prepare a nanofibrous membrane chemical sensor with superior stability. The GQDs-PAN nanofibrous membrane exhibited irreversible fluorescence quenching effect and excellent selectivity under free active chlorine treatment. The GQDs-PAN nanofibrous membrane exhibited better photostability and lower detection limit than the single GQDs solution, also had good luminescence performance under light irradiation for 2 months and the detection limit for hypochlorite was as low as 2 μM. Based on its excellent stability and low detection limit, it has a good application prospect in practical environmental monitoring (Fig. 21(A)).

### Electrospun nanofiber sensor for detection of SO_3_^2−^

Gao *et al.*^131^ prepared a CNFs/MnCo_2_O_4.5_ composite fiber membrane material by electrospinning technology and electrochemical deposition technology. The fiber membrane can be used for the colorimetric detection of sulfite, showing a good linear relationship in the sulfite concentration range of 0–1 μM, and the detection limit was calculated to be 15.9 nM. Its excellent colorimetric effect and high sensitivity enable the application of manganese cobalt oxide/carbon nanofiber composites in chemical sensing (Fig. 21(B), (C) and (D)).

Cyanide, as a strong electron-withdrawing ion, can achieve sensing by forming strong interactions with active N–H and phenolic hydroxyl groups to deprotonate them, resulting in changes in fluorescence and color. It is also possible to use the etching effect of cyanide on the metal to cause the change of fluorescence to realize the sensing of the reinforcement. Hypochlorite and sulfite except according to the inorganic sensing unit described above. It is also possible to use the oxidation of hypochlorite to design some organic dyes containing sulfur and selenium as sensing units, or to use the nucleophilicity of sulfites to design organic dyes containing carbon positive structure as sensing units.

## Conclusion and future prospects

In this paper, we summarize and compare the recent advances in electrospun fiber membrane chemical sensors in the field of ion detection in the past decade (Table 1). The preparation of electrospun fiber membrane sensors is a unit that will be able to act as sensing through physical doping, chemical modification, copolymerization, surface adsorption and self-assembly, such as organic molecular probes, MOFs, carbon quantum dots, noble metal nanoparticles and natural biomaterials *etc.* are immobilized on the electrospun fibers.

**Table 1:** Comparison of materials and properties of fiber membrane sensors

Analyte	Fiber material	Sensing unit	Preparation	Sensor types	LOD	Repeatability	Ref.
Cu^2+^	PES	Rhodamine derivatives	Physical doping	Fluorescence/colorimetric	1.1 nM	Yes	79
Cu^2+^	CS/PAN	Rhodamine derivatives	Physical doping	Colorimetric	10^−8^ M	Yes	80
Cu^2+^	PMMA	RB	Physical doping	Fluorescence/colorimetric	0.11 μM/0.28 μM	Yes	81
Cu^2+^/Cr^3+^	CA	1,4-DHAQ	Physical doping	Fluorescence	3 × 10^−9^ M	Yes	82
Cu^2+^	Poly (NIPAAm-*co*-NMA)	F-phen	Physical doping	Fluorescence	—	No	83
Cu^2+^	PMMA	PEI-EAS NPs	Physical doping	Fluorescence	—	Yes	84
Cu^2+^	PMAR	Rhodamine B-hydrazine	Chemical modification	Fluorescence/colorimetric	1.5 × 10^−6^ M	Yes	85
Cu^2+^/Hg^2+^	P(HEMA-*co*-NMA)	PyDAN2/RhBN2	Chemical modification	Fluorescence	10^−7^ M/10^−6^ M	Yes	86
Cu^2+^	PVA	L	Chemical modification	Colorimetric	1.27 × 10^−8^ M	No	87
Cu^2+^	aPAN	BPEI	Chemical modification	Colorimetric	11.5 μM/4.8 μM	No	88
Cu^2+^	aPAN	Au/Ag NPs	Chemical modification	Colorimetric	50 nM	Yes	89
Cu^2+^	PNNR	RHPMA	Copolymerization	Fluorescence	—	Yes	90
Cu^2+^	PEI/PVA	CdTe-QDs	Surface adsorption	Fluorescence	11.1 nM	No	91
Cu^2+^	CA	DTT.AuNC	Surface adsorption	Fluorescence	16 μM	No	92
Fe^3+^	EC	CBINH	Physical doping	Fluorescence	7 × 10^−14^ M	Yes	93
Fe^3+^	PMMA	1/1A	Physical doping	Fluorescence	174 ppb/59 ppb	No	94
Fe^3+^	PCL	FPN	Physical doping	Fluorescence	2.9413 nM	No	95
Fe^3+^	PAN	Eu-MOF	Physical doping	Fluorescence	63 nM	No	97
Fe^3+^	PAN	1-Eu^3+^	Physical doping	Fluorescence	6.685 × 10^−4^ M	No	98
Fe^3+^	Zein	Curcumin	Physical doping	Colorimetric	0.4 mg L^−1^	No	99
Fe^3+^	PPy-*b*-PNIPAAm-*b*-PNMA	PPy	Chemical modification	Fluorescence	—	No	102
Fe^3+^	PANA	Coumarin derivatives	Chemical modification	Fluorescence	10.63 μM	No	103
Fe^3+^	PASP	PASP	Chemical modification	Colorimetric	0.1 mg L^−1^	No	104
Fe^3+^	Poly (MMA-*co*-RQ)	RQ	Copolymerization	Colorimetric	1.19 μM	Yes	105
Hg^2+^	PVA	AuCN	Physical doping	Fluorescence	1 ppb	No	106
Hg^2+^	Polyurethane	RIM	Physical doping	Fluorescence	—	No	107
Hg^2+^	PMMA	RBH/R6GH	Physical doping	Fluorescence/colorimetric	0.8 ppb/3.6 ppb	Yes	108
Hg^2+^	Poly(NIPAAm-*co*-NMA-*co*-AA)	BNPTU	Physical doping	Fluorescence	—	No	109
Hg^2+^	PEO/CS	CQDs	Physical doping	Fluorescence	—	Yes	110
Hg^2+^	BSA/PEO	Au–NCs	Chemical modification	Fluorescence	57 pM	No	111
Hg^2+^	PAN	PAR	Chemical modification	Colorimetric	35 μg L^−1^	No	112
Hg^2+^	P(NIPAAm-*co*-NMA-*co*-RhBN2AM)	RhBN2AM	Copolymerization	Fluorescence	—	Yes	113
Hg^2+^	PAN	DTPDI	Surface adsorption	Colorimetric	1 ppb	No	114
Hg^2+^	N6/FITC	Au@BSA	Surface adsorption	Fluorescence	1 ppt	No	115
Hg^2+^	PCL-NF	AuNC	Surface adsorption	Fluorescence	ppt level	No	116
Hg^2+^	Poly(MMA-*co*-ADMA)	Thiocarbamido-SRhB-β-CD	Self-assembly	Fluorescence/colorimetric	6.0 × 10^−5^ mol L^−1^	No	117
Pb^2+^	CA	Curcumin	Physical doping	Colorimetric	20 μM	No	118
Pb^2+^	DCA	PMAD	Chemical modification	Colorimetric	0.048 mM	Yes	119
Pb^2+^	Bacterial cellulose	Curcumin	Surface adsorption	Colorimetric	9 μM/0.9 μM	No	120
Pb^2+^	PA-6/NC	BAu probe	Surface adsorption	Colorimetric	0.2 μM	Yes	121
Al^3+^	Polyurethane	R2PP	Physical doping	Fluorescence	8.5 × 10^−9^ M	Yes	122
Zn^2+^	PHEM	TPN-Cl2	Physical doping	Fluorescence	—	No	123
Zn^2+^	Poly (MMA-*co*-Sal)	HAB	Copolymerization	Fluorescence	1.95 × 10^−5^ mol L^−1^	No	124
CN^−^	PVA@CA	CDs/AuNC	Physical doping	Fluorescence	0.15 μM	No	125
CN^−^/F^−^	Polyurethane	M	Physical doping	Fluorescence/colorimetric	—	No	126
CN^−^	PMMA/EHCE	PBM	Chemical modification	Fluorescence	2.15 × 10^−5^ M	No	127
F^−^	PAN	AgPt–Fe_3_O_4_@SiO_2_	Physical doping	Colorimetric	3.73 μM	No	128
F^−^	PAN-*co*-AA/β-CD	Intermediate 3	Self-assembly	Fluorescence	—	No	129
ClO^−^	PAN	GQDs	Physical doping	Fluorescence	2 μM	No	130
SO_3_^2−^	CNFs	MnCo_2_O_4.5_	Chemical deposition	Colorimetric	15.9 nM	No	131

In conclusion, electrospun nanofiber membranes for ion detection can be prepared using physical doping, copolymerization, chemical modification, self-assembly and surface adsorption. Among them, the physical doping method is the simplest and the widest range of use, but there are also shortcomings such as low utilization rate of the sensing unit and easy separation of the sensing unit from the polymer. The copolymerization method enables the sensing unit and the polymer to be tightly bonded, but the low utilization of the sensing unit is not improved. Chemical modification, surface adsorption and self-assembly can solve the above problems and improve the utilization rate of the sensing unit while making the combination more stable. This provides some ideas for the development of ion electrospun fiber sensors. The electrospun fiber membrane sensor can rapidly and sensitively detect metal ions and anions in water by colorimetric-fluorescence, due to the large specific surface area and high permeability of the fiber membrane and most of the fiber membrane sensors also have good regeneration ability. It is not easy to cause pollution in the process of detecting harmful substances and it has the characteristics of environmental friendliness, because solid-state sensors can be easily separated from water.

**Scheme 2:**
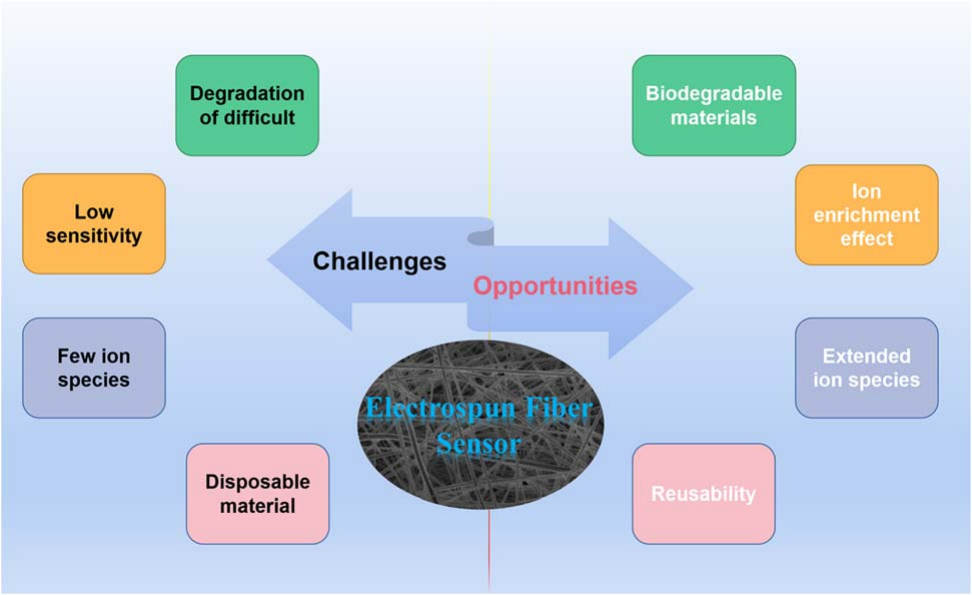
The challenges and opportunities of electrospun fiber membrane ion sensor.

However, there are some challenges in the development of electrospun membrane sensors. At present, most of the electrospun fibers use polymers that are not easy to degrade, which are easy to form microplastics in the environment. The pollution of microplastics has become a major environmental problem in today's world. Although electrospun membrane sensors have a very high specific surface area and porosity, the heterogeneous system formed with water limits the contact between the detected ions and the sensor itself. Therefore, the detection limit of the sensor is still relatively high, and the detection of trace ions cannot be completed. In addition, the current electrospun fiber membranes for ion detection mainly focus on the detection of metal ions, but for the case of water pollution, some electrospun fiber membranes for common highly toxic anion detection have not been reported. The reusability of the sensor is an important indicator. However, only a very small number of electrospun fiber membrane sensors have been reported to study their reusability (Scheme 2).

At the same time as the challenges, there are many opportunities for electrospun membrane sensors. There are many polymeric materials with good degradability. Examples include chitosan, cellulose, protein and amino acid polymers.^132^ These polymeric materials can be decomposed by microorganisms in the environment into environmentally friendly inorganic substances such as carbon dioxide, nitrogen and water. Moreover, in order to improve the detection sensitivity of nanofiber membrane sensors to various ions, electrochemical methods can be introduced to improve the sensing sensitivity. Alternatively, a fiber membrane material that can enrich ions in water can be designed based on the electrostatic interaction of anions and cations so that low concentrations of ions can also respond to the fiber membrane sensor.^133–135^ Expanding the detection of ion types of sensors is also the future development direction of electrospun fiber membrane sensors. Some anions containing arsenic and chromium in the environment pose a serious threat to human health and the environment. It is necessary to design fiber membranes that can efficiently detect and remove these ions. Finally, for the sustainable development of electrospun membrane sensors, exploring their reusability is also a highlight.

## Author contributions

Liangqiang Wu: conceptualization; writing–original draft; investigation, Yan Song: writing – review & editing; supervision, Shuo Xing: resources; investigation, Yapeng Li: resources; writing – review & editing, Hai Xu: writing – review & editing, Qingbiao Yang: writing – review & editing; funding acquisition, supervision, Yaoxian Li: supervision; writing – review & editing.

## Conflicts of interest

There are no conflicts to declare.

## Supplementary Material
